# Correlates of irregular family meal patterns among 11-year-old children from the Pro Children study

**DOI:** 10.1080/16546628.2017.1339554

**Published:** 2017-06-22

**Authors:** Torunn Holm Totland, Markus Dines Knudsen, Mari Mohn Paulsen, Mona Bjelland, Pieter van’t Veer, Johannes Brug, Knut Inge Klepp, Lene Frost Andersen

**Affiliations:** ^a^ Department of Nutrition, Institute of Basic Medical Sciences, University of Oslo, Oslo, Norway; ^b^ Division of Human Nutrition, Wageningen University, Wageningen, The Netherlands; ^c^ Amsterdam School of Communication Research, University of Amsterdam, Amsterdam, The Netherlands; ^d^ Department of Mental and Physical Health, Norwegian Institute of Public Health, Oslo, Norway

**Keywords:** Irregular family meals, children, social differences, fruit and vegetable intake, screen time

## Abstract

**Background:** The importance of family meals to the consumption of healthful food choices has been stated in recent reviews. However, little information is available on barriers that interfere with regular family meal patterns during childhood.

**Objective:** Describe family meal patterns among 11-year-old children across Europe and identify correlates of irregular family breakfast and dinner consumption.

**Design:** Cross-sectional survey involving samples of 13,305 children from nine European countries in 2003.

**Results:** The proportions of children who regularly ate family breakfast and dinner were 62% and 90%, respectively. Correlates of irregular family breakfasts and dinners were less vegetable consumption, and irregular family breakfasts were associated with more television viewing. Social differences in the consumption of family breakfasts were observed.

**Discussion:** Strengths of this study are the large sample size and validated research method. Limitations are the cross-sectional design and self-reported data.

**Conclusion:** The majority of 11-year-old children regularly ate breakfast and dinner with their families. Less vegetable consumption and more television viewing were associated with irregular family breakfasts and dinners, respectively. Social differences were observed in the regularity of family breakfasts. Promoting family meals across social class may lead to healthier eating and activity habits, sustainable at the population Level.

## Background

The establishment of healthy eating behaviours at an early age is important given that these behaviours tend to continue into adulthood [1]. Early adolescence is a period in which parents still play a central role in influencing their children’s food choices and future eating habits [2]. Family meals are considered an important arena for parents to socialize their children, as well as act as role models and monitor their children’s behaviours [3,4]. The promotion of healthy family meals within this age group may therefore result in healthy food choices throughout the life course [5].

**Figure 1.**
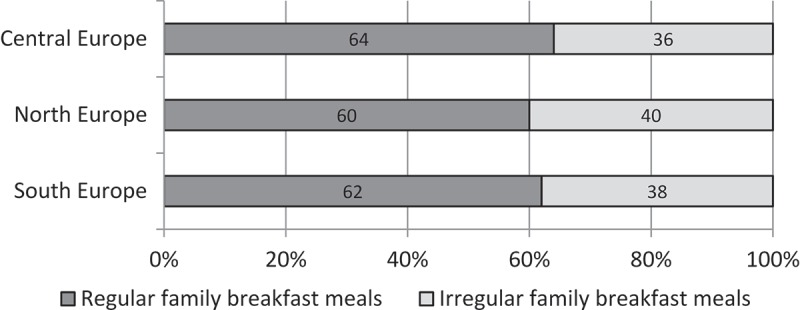
Prevalence of family breakfast meals in Europe (*n* = 12,880).

**Figure 2.**
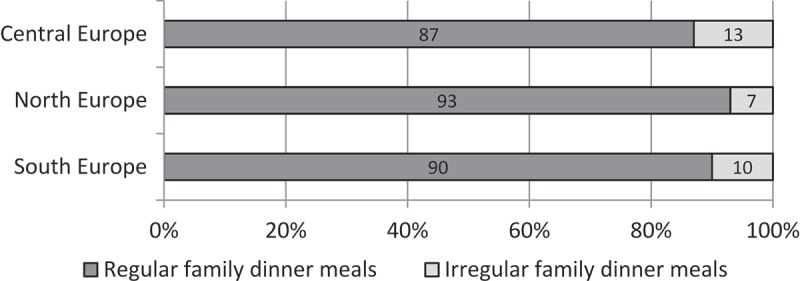
Prevalence of family dinner meals in Europe (*n* = 12,807).

Recent reviews have found consistent evidence of a favourable association between engagement in family meals and weight status outcomes among children and adolescents [6,7]. However, the majority of this evidence is based on cross-sectional studies, and further longitudinal studies are needed [6–8]. Studies have also found that family meals are associated with better school performance [9,10], higher levels of emotional well-being [9,11], fewer weight control issues [11], and reduced risk behaviours [12] among children and adolescents.

The importance of family meals for more healthful food choices has also been stated in recent reviews [6,7]. More frequent family meals have been associated with higher intake of fruits, vegetables and key nutrients, as well as lower intake of soft drinks and saturated fat and a lower number of skipped meals during childhood and into adulthood [13–16]. However, other studies have been unable to show any direct association between the frequency of family meals and the intake of fruits and vegetables in late adolescence [17,18].

Despite the posited and partly evidence-based positive health outcomes associated with family meals, little information is available regarding which children are more or less likely to engage in family meals, i.e. the correlates of irregular meal patterns in late childhood or adolescence [19,20]. In general, a higher socioeconomic position has been associated with healthier food choices [21,22]. The skipping of breakfast has been associated with the female gender, later adolescence, living in single parent families [23], and lower socioeconomic position [23,24]. Increased breakfast skipping has also been found to be associated with less physical activity and more television viewing [23].

The prevalence of family meals has been found to vary across European countries among children between the ages of 10 and 12 and is more prevalent in the northern countries than in southern and eastern countries [25]. However, data on family meals are scarce in Europe, and nationally representative data across Europe is not currently available [7,25,26]. The aim of the present study was to describe family meal patterns among 11-year-old children across Europe and identify correlates of irregular family breakfast and dinner consumption in different regions of Europe.

## Materials and methods

### Study sample

As part of the Pro Children project, a cross-sectional study was carried out in nine different European countries (Austria, Belgium, Denmark, Iceland, the Netherlands, Norway, Portugal, Spain, and Sweden) between October and December in 2003. Nationally representative samples were drawn in each country except for Austria, which was represented by the eastern region covering 42% of the total population, and Belgium, which was represented by the Flanders. Eleven-year-old children born in 1992 were the target group, but the survey involved children between the ages of 9 and 14 years. A total of 15,404 school children were asked to complete a self-administered paper-based questionnaire. The questionnaire was developed in English, then translated into the children’s native languages and finally back-translated to English before being returned to the research centre. The translators were proficient and not involved in the development of the questionnaire.

Written consent was obtained from the parents prior to data collection, and ethical clearance was obtained from the research ethics committees of each of the nine countries [27]. The questionnaire was completed by the children at school during class hours and administered by the teacher. An inspiration and training session was arranged for the involved teachers in the beginning of the project. The survey was anonymous in all of the countries. A total of 13,924 children returned questionnaires, of which 110 were unanswered and 144 were destroyed at the parent’s request. An additional 306 questionnaires were excluded because of unreliable answers, and 59 were excluded because of missing gender information. The final sample included in this study consisted of 13,305 children (86%) with complete questionnaires. The rationale, design, and methodology of the Pro Children study has been described in more detail elsewhere [27].

### Questionnaire

In the questionnaire, the children were asked the following two questions about family meals: (1) How often do you have breakfast with your mother and/or father? and (2) How often do you have dinner (supper/evening meal) with your mother and/or father? The answer categories included the following: every day, 4–6 days a week, 1–3 days a week, less than 1 day a week, and never. How often the children were eating with their parents was dichotomized into regular (4 days a week or more) and irregular (less than 4 days a week) categories for both family meal variables.

The parents’ occupations were also child-reported and based on the following questions: (1) Does your mother have a job (yes/no)? and (2) Does your father have a job (yes/no)? Both questions were followed up with two open-ended questions: (3) If YES, please write in what place she/he works, and (4) please write which job she/he has there. We coded the registered occupations from the questionnaires into five categories of social class (1 = high, 5 = low) based on the standards of the Danish National Institute of Social Research [28]. Parents categorized as ‘in education’, ‘without paid job’, and ‘not defined’ were defined as social class 6. For the purpose of this paper, the child’s social class was defined by the parent with the highest rated occupation. If information regarding the occupation of one parent was missing, the information from the other parent was used. The social class variables were regrouped into four categories: high (social classes 1 and 2), medium (social class 3), low (social classes 4 and 5), and other (social class 6).

Differences in the prevalence of obesity and weight-related behaviours, including the prevalence of family meals, have been found when comparing the northern and southern regions of Europe [24,25]. Participants from the nine European countries were therefore regrouped into the following three geographical regions in order to explore such differences: central (Austria, Belgium, the Netherlands), north (Denmark, Iceland, Norway, Sweden), and south (Portugal, Spain).

The children were asked how often they ate fresh fruit, salad or grated vegetables, other raw vegetables, potatoes, cooked vegetables, and fruit juice. The answer categories were the following: never, less than 1 day per week, one day per week, 2–4 days per week, 5–6 days per week, once a day, twice a day, and more than twice a day. As previously described by Kristjansdottir et al. [29], the frequencies were recoded in units of grams per day according to the standards supported by the WHO [30]. One portion of fresh fruit equals 100 g, one portion of salad equals 40 g, one portion of raw vegetables equals 50 g, and one portion of cooked vegetables equals 60 g [29]. A total fruit variable was defined as the usual intake of fresh fruit, and a total vegetable variable was defined as the usual intake of salad or grated vegetables, other raw vegetables, and cooked vegetables.

Leisure-time physical activity was assessed by the question ‘Outside school hours: how many hours a week do you usually exercise in your leisure time so much that you get out of breath or sweat?’ The answer categories were the following (recoded into hours/week, as in brackets): none (0), about half an hour a week (0.5), approximately 1 hour a week (1), approximately 2–3 hours a week (2.5), approximately 4–6 hours a week (5), and 7 hours a week or more (7). Leisure-time television viewing was assessed by the question ‘About how many hours a day do you watch television and videos in your leisure time?’ Similarly, leisure time spent on a computer was measured by the question ‘About how many hours a day do you usually use a computer (for playing games, emailing, chatting or surfing the internet) in your leisure time?’ The answer categories were the following (recoded into hours/day, as in brackets): none (0), about half an hour a day (0.5), approximately 1 hour a day (1), approximately 2 hours a day (2), approximately 3 hours a day (3), approximately 4 hours a day (4), approximately 5 hours a day (5), approximately 6 hours a day (6), and approximately 7 hours a day or more (7). The children were also asked the following question about television during meal times ‘How often is the television on during dinner (supper/evening meal) in your home? The answer categories were (recoded into frequency/week, as in brackets) never (0), less than 1 day a week (0.5), 1–3 days a week (2), 4–6 days a week (5), and every day (7).

### Statistical analyses

Descriptive statistics on the children’s characteristics for all variables are presented as proportions or group means with corresponding standard deviations. The distributions of the children’s characteristics based on the regularity of family breakfasts and dinners are described in detail for each region of Europe. Multilevel analyses were considered because the data were hierarchical, and in theory, individuals within a defined group were expected to be more similar to each other than the other groups [31,32]. However, as only 1% of the unexplained variance in the dependent variables was shown to be at the group (i.e. school class) level, multilevel analyses were not performed. It has also been shown that the eating habits of pupils’ appear to be largely independent of school [33–35], whereas interpersonal factors play a more important role [33]. Multivariate logistic regression analyses were conducted in the stratified analysis of each European region with the regularity of family breakfasts and dinners as the dependent variables. The models were adjusted for all the potential correlates analysed including gender, social class, fruit intake, vegetable intake, physical activity, television time, and computer time. All analyses were performed using IBM® SPSS® Statistics, version 20.0 (IBM Corporation).

## Results

The descriptive statistics of the participating European 11-year-old children are presented in Table 1.

**Table 1.** Descriptive characteristics of European 11-year-old children (*n* = 13,305).

Variable	Measure	n*	Mean	SD	%
Gender	Total	13,305			100
	Girl	6625			50
	Boy	6680			50
European part	Total	13,305			100
	Central	4140			31
	North	5718			43
	South	3447			26
Social class	Total	13,050			100
	High	3164			24
	Med	2865			22
	Low	5543			43
	Other	1478			11
Family breakfast	Total	12,880			100
	Regular	7974			62
	Irregular	4906			38
Family dinner	Total	12,807			100
	Regular	11,546			90
	Irregular	1261			10
Age	Years	13,238	11.3	0.6	
Fruit intake	Gram/day	13,168	101.2	85.9	
Vegetable intake	Gram/day	12,905	59.0	51.6	
Physical activity	Hour/week	12,985	2.8	2.2	
Television time	Hour/week	12,773	2.3	1.7	
Computer time	Hour/week	12,917	1.2	1.4	
Television dinner	Freq/week	12,949	3.4	3.0	

*Number of cases (*n*) varies slightly across variables.

The proportion of participating children was evenly distributed between both genders, and the mean age was 11 years. The proportion of children representing the different regions of Europe was highest in the northern region (43%) and lowest in the southern region (26%) due to the number of participating countries. Almost half of the children were characterized as low social class (43%). The proportion of children who regularly consumed family breakfasts was 62% across Europe, with proportions of 60% in the northern region, 62% in the southern region, and 64% in central Europe (Figure 1).

The proportion of children who regularly consumed family dinners was 90% across all countries, with proportions 87% in the central region, 90% in the southern region, and 93% in the northern region of Europe (Figure 2).

The descriptive characteristics of family breakfast consumption are presented in Table 2.

**Table 2.** Regularity of family breakfast meals in Europe.

		Central Europe		North Europe		South Europe	
		Regular	Irregular	Regular	Irregular	Regular	Irregular
Variable	Measure	(*n*=2599*)	(*n*=1457*)	(*n*=3321*)	(*n*=2187*)	(*n*=2054*)	(*n*=1262*)
Gender (%)	Girl	49	54	48	51	48	53
	Boy	51	46	52	49	52	47
Social class (%)	High	28	20	30	20	20	22
	Med	24	27	24	22	18	16
	Low	34	41	35	45	56	56
	Other	14	12	11	13	6	6
Age (y)	Mean	11	11	11	11	11	11
	SD	1	1	1	1	1	1
Fruit (g/d)	Mean	102	93	104	87	117	102
	SD	83	83	88	81	89	86
Vegetable (g/d)	Mean	67	56	60	48	63	54
	SD	49	45	53	44	58	54
Physical activity (h/w)	Mean	3.1	2.9	3.3	3.0	2.0	2.0
	SD	2.2	2.2	2.2	2.3	2.0	2.0
Television time (h/w)	Mean	2.4	2.7	2.0	2.3	2.4	2.6
	SD	1.7	1.8	1.4	1.6	1.8	1.9
Computer time (h/w)	Mean	1.3	1.4	1.0	1.1	1.1	1.1
	SD	1.5	1.5	1.3	1.4	1.5	1.5

*Number of cases (*n*) varies slightly across variables.

The proportion of girls consuming irregular family breakfasts ranged from 51% in northern to 54% in central Europe. Children from lower social classes were more likely to be irregular family breakfast consumers in both the central and northern regions of Europe. Fruit intake was lower among irregular family breakfast consumers than regular consumers in all regions of Europe and ranged from 87 g per day in the northern region to 102 g per day in the southern region. Vegetable intake was consistently lower among irregular family breakfast consumers than more regular consumers. Leisure time spent watching television was higher among irregular consumers of family breakfast and ranged from 2.3 hours per week in the northern region to 2.7 hours per week in the central region of Europe.

The descriptive characteristics of family dinner consumption are presented in Table 3.

**Table 3.** Regularity of family dinner meals in Europe.

		Central Europe		North Europe		South Europe	
		Regular	Irregular	Regular	Irregular	Regular	Irregular
Variable	Measure	(*n*=3501*)	(*n*=517*)	(*n*=5087*)	(*n*=400*)	(*n*=2958*)	(*n*=344*)
Gender (%)	Girl	51	47	50	47	51	43
	Boy	49	53	50	53	49	57
Social class (%)	High	26	19	27	16	21	22
	Med	25	27	23	23	17	14
	Low	36	39	38	47	56	58
	Other	13	15	12	14	6	6
Age (y)	Mean	11	11	11	11	11	11
	SD	1	1	1	1	1	1
Fruit (g/d)	Mean	99	95	98	80	113	100
	SD	82	87	86	78	88	89
Vegetable (g/d)	Mean	65	52	57	43	60	53
	SD	47	46	51	38	56	57
Physical activity (h/w)	Mean	3.1	2.9	3.2	2.7	2.0	2.2
	SD	2.2	2.2	2.3	2.3	2.0	2.3
Television time (h/w)	Mean	2.5	2.7	2.1	2.4	2.4	2.8
	SD	1.7	1.9	1.5	1.6	1.8	2.1
Computer time (h/w)	Mean	1.3	1.5	1.1	1.2	1.1	1.4
	SD	1.4	1.7	1.3	1.5	1.5	1.9
Television dinner (f/w)	Mean	2.7	3.4	2.8	3.5	5.3	4.8
	SD	2.8	2.9	2.8	2.8	2.6	2.8

*Number of cases (*n*) varies slightly across variables.

Contrary to what was observed for family breakfasts, the proportion of irregular family dinner consumption was consistently higher among boys than girls, ranging from 53% in the central and northern regions to 57% in the southern region of Europe. The differences according to social class were similar to what was observed for family breakfasts, with children from lower classes being more likely to have irregular family dinners. Fruit and vegetable intake was also lower among irregular consumers of family dinners than regular consumers in all regions of Europe. Leisure time spent watching television or on a computer was consistently higher among irregular consumers of family dinners across Europe. The frequency of television viewing during dinnertime among irregular consumers ranged from 3.4 times per week in the central region to 4.8 times per week in the southern region.

The potential correlates of irregular family breakfast consumption based on the multivariate logistic regression models in the different regions of Europe are shown in Table 4.

**Table 4.** Odds ratios (95% confidence intervals) for potential correlates of an irregular family breakfast meal consumption (*n* = 11,739).

	Central Europe (*n*=3799)		North Europe (*n*=4900)		South Europe (*n*=3040)	
Potential correlates	OR	CI	OR	CI	OR	CI
Gender (boy vs. girl*)	0.73***	0.64–0.85	0.80***	0.70–0.90	0.78**	0.67–0.91
Social class (medium vs. high*)	1.53***	1.26–1.86	1.35**	1.14–1.61	0.71**	0.56–0.91
Social class (low vs. high*)	1.60***	1.34–1.92	1.77***	1.52–2.06	0.81**	0.67–0.99
Social class (other vs. high*)	1.19	0.94–1.51	1.60***	1.30–1.96	0.81	0.57–1.14
Fruit intake (per 50g/d)	0.99	0.95–1.04	0.94**	0.91–0.98	0.94**	0.89–0.98
Vegetable intake (per 50g/d)	0.77***	0.70–0.83	0.84***	0.78–0.90	0.89**	0.83–0.96
Physical activity (h/w)	0.99	0.97–1.03	0.98	0.95–1.00	1.00	0.96–1.04
Television time (h/w)	1.09***	1.04–1.14	1.09***	1.04–1.13	1.08***	1.03–1.12
Computer time (h/w)	1.03	0.98–1.08	1.03	0.98–1.08	0.96	0.91–1.02

*Reference group.

Statistical significance at **p < 0.05 and ***p < 0.001.

All analyses are adjusted for all potential correlates.

Boys had significantly lower odds of irregular family breakfast consumption than girls in all parts of Europe, ranging from 27% lower odds in the central region to 20% lower odds in the northern region. In the central and northern regions of Europe, the odds of being an irregular breakfast consumer increased with lower social class and were significantly higher among those in the medium and low social classes when compared with those in the high social class. The opposite was observed in the southern region of Europe, where lower social class was associated with significantly lower odds of being an irregular breakfast consumer compared with children from higher social classes. The odds of fruit and vegetable intake were significantly lower in irregular family breakfast consumers than in regular consumers in all regions, except for fruit intake in the central region of Europe where no significant differences were observed. The odds of time spent watching television were significantly higher among irregular family breakfast consumers, compared to regular consumers in all European regions.

The potential correlates of irregular family dinner consumption in the different regions of Europe based on the multivariate logistic regression models are presented in Table 5.

**Table 5.** Odds ratios (95% confidence intervals) for potential correlates of an irregular family dinner meal consumption (*n* = 11,648).

	Central Europe (*n*=3766)		North Europe (*n*=4876)		South Europe (*n*=3006)	
Potential correlates	OR	CI	OR	CI	OR	CI
Gender (boy vs. girl*)	1.06	0.86–1.29	1.03	0.82–1.30	1.12	0.87–1.45
Social class (medium vs. high*)	1.21	0.91–1.61	1.50**	1.06–2.14	0.71	0.47–1.09
Social class (low vs. high*)	1.20	0.91–1.56	1.59**	1.16–2.19	1.04	0.76–1.42
Social class (other vs. high*)	1.31	0.94–1.81	1.67**	1.12–2.49	1.06	0.61–1.84
Fruit intake (per 50g/d)	1.06	1.00–1.13	0.93	0.86–1.01	0.93	0.86–1.00
Vegetable intake (per 50g/d)	0.69***	0.61–0.79	0.82**	0.70–0.95	0.88	0.77–1.00
Physical activity (h/w)	0.99	0.94–1.03	0.94**	0.89–0.99	1.02	0.96–1.08
Television time (h/w)	1.01	0.95–1.08	1.07	1.00–1.15	1.09**	1.02–1.16
Computer time (h/w)	1.03	0.96–1.11	1.02	0.94–1.11	1.13**	1.05–1.22
Television dinner (f/w)	1.07***	1.03–1.11	1.05**	1.01–1.10	0.90***	0.86–0.94

*Reference group.

Statistical significance at **p < 0.05 and ***p < 0.001.

All analyses are adjusted for all potential correlates.

In the northern region, children in low, medium, and other social classes were more likely to have irregular family dinner consumption compared with those in high social classes. However, these differences were not statistically significant in the central and southern regions of Europe. The odds of having a higher vegetable intake were significantly lower among irregular family dinner consumers than regular consumers in the central (31% lower odds) and northern regions (18% lower odds). In the northern region, irregular family meal consumers had 6% lower odds of regular physical activity than regular consumers. However, such a difference was not observed in the central and southern regions of Europe. Time spent watching television or on computers had 9% and 13% higher odds, respectively, among the irregular family dinner consumers in the southern region of Europe compared with regular consumers. Television viewing during dinner had 7% and 5% higher odds among the irregular family dinner consumers in the central and northern regions of Europe, respectively, compared with regular consumers. However, in the southern region of Europe, children engaging in irregular family dinner consumption had 10% lower odds of watching television during dinner compared with those engaging in regular family dinner consumption.

## Discussion

The results from the present study showed a high prevalence of family dinner consumption among 11-year-old children across Europe, but a lower prevalence of family breakfast consumption was observed. Irregular family breakfasts were more prevalent in the northern region when compared to the southern and central regions of Europe, and irregular family dinners were more prevalent in the central region of Europe when compared to northern and southern regions. Irregular family breakfasts and dinners were found to be associated with less vegetable consumption, and irregular family breakfasts were associated with more television viewing. Moreover, social class differences were observed in the regularity of family breakfasts in most of the European countries, with children from lower classes being more likely to engage in irregular meal patterns.

In the ENERGY (EuropeaN Energy balance Research to prevent excessive weight Gain among Youth) cross-sectional study of 7716 children (10–12 years old) from eight European countries, the prevalence of regular family breakfasts (35%) and dinners (76%) was lower than the results from the present study [36]. There is scarce knowledge regarding the prevalence of family meals from a pan-European perspective. However, several pan-European studies of breakfast habits among children and adolescents exist. Our results differ from previous cross-sectional findings regarding family breakfasts in which family breakfast consumption was found to be more prevalent in the northern region of Europe when compared to the southern and eastern regions [25]. Regarding family dinners, our results correspond to previous results that showed higher proportions of family dinner consumers in the northern region compared with the southern and eastern regions of Europe [25]. In the ENERGY project, the regularity of daily breakfast consumption among children varied widely across countries. The number of days in which breakfast was consumed per week was highest in Spain, Norway, the Netherlands and Belgium and lowest in Greece and Hungary [24,37]. Children living in the Netherlands, Belgium, and Norway also showed a more favourable pattern regarding the personal variables (knowledge, preferences, attitude, and health beliefs) of breakfast consumption, whereas children from Hungary and Slovenia had a less favourable pattern [38].

In the present study, we observed significantly higher odds of irregular family breakfast consumption among girls than boys in the northern, central, and southern regions of Europe. The same tendency was observed among almost 3000 middle and high school students in the Minneapolis-St. Paul area where family breakfast frequency was found to be positively associated with the male gender [39,40].

The odds of having a higher vegetable intake were lower among irregular consumers of both family breakfasts and dinners compared with regular consumers in all parts of Europe. With regard to fruit consumption, the odds of having a higher intake were significantly lower among irregular family breakfast consumers in the northern and southern regions of Europe, but no significant differences were observed in the central region. In the study from the Minneapolis-St. Paul area, family breakfast frequency was also positively associated with the intake of fruits [39,40] and vegetables [40] among adolescents. In that study, adolescents who reported eating a family breakfast every day consumed an average of 0.37 more daily fruit servings than adolescents who did not consume any family breakfasts [39]. It has been argued that the food eaten during family meals may vary between countries due to differences in food culture. Therefore, having frequent family meals may be an indicator of a healthy family lifestyle in the family only some countries [25].

We found that irregular family breakfast consumption was associated with more time spent watching television in all parts of Europe. Irregular family dinner consumption in the southern region of Europe was associated with more time spent watching television and on the computer. However, such an association was not observed in the central and northern regions of Europe. Irregular family dinner consumption in the central and northern regions of Europe was associated with watching television during dinner, whereas the opposite was observed in the southern region where irregular family dinner meal consumption was associated with lower odds of television viewing during dinner. A positive association between having a television on during family meals and obesity has previously been described among children in the northern region of Europe but not in the southern and eastern regions [25]. Among adolescents in the Minneapolis-St. Paul area of the USA, watching television during family meals was found to be associated with lower intake of vegetables and higher intake of soft drinks compared with adolescents who did not watch television during meals [41]. Previous studies have shown that television viewing among children is associated with the consumption of more unhealthy foods in both Europe [42] and America [43]. The foods and beverages consumed while watching television by children and adolescents who watch more than 2 hours per day have been found to be more energy dense than the foods consumed by children and adolescents who watch less television [42,44–46]. In a study involving eight European countries, the authors found that those who ate breakfast while watching TV were more likely to be obese [47].

Our results suggest that children from lower social classes in the central and northern regions of Europe were more likely to be irregular consumers of family breakfasts compared with children from higher social classes. These findings correspond with the results of the ENERGY study in which children with highly educated parents were more likely to have family breakfasts than children with less educated parents [36]. Several earlier studies have also indicated that children who live in families with low socioeconomic status (SES) are more likely to have irregular breakfast consumption [23,37,48–50]. Research from Minneapolis/St. Paul has found that more frequent family meals are associated with higher SES [40].

The main strength of the Pro Children study is the large sample size representing all regions of Europe. The research method used in this study has been validated with a satisfactory measure of intake, attitudes and preferences [51]. A major limitation of cross-sectional surveys such as Pro Children is the limited opportunity to comment on the causality between consuming family meals and determinants. We can only describe the correlates associated with family meals. Another limitation is the self-reporting data from the children, particularly regarding their parents’ occupations and social class. In the Pro Children survey, parental questionnaires were also used to measure parental social class. However, many of the parents did not complete the questionnaire. Hence, the data regarding SES from the parents were more deficient than the data from the pupils. The preciseness of these data may have influenced the results. Moreover, the data from the Pro Children study were collected 13 years ago, which may affect the relevance for today’s school children. However, the proposed research questions are still highly relevant, and the current data brings light to social differences in family meals across Europe based on available national representative data.

## Conclusion

Data from the Pro Children study suggest that the majority of 11-year-old children regularly eat breakfast and dinner with their families. However, a substantial group has an irregular family breakfast pattern, which is more common in northern than central and southern Europe. Irregular family breakfasts and dinners are associated with less vegetable consumption, and irregular family breakfasts are associated with more television viewing. Moreover, social differences exist in the regularity of family breakfasts in most European countries, and the promotion of family meals across social class may lead to healthier eating and activity habits that are sustainable at the population level.
